# Location- and object-based attention enhance number estimation

**DOI:** 10.3758/s13414-020-02178-w

**Published:** 2020-11-06

**Authors:** Antonella Pomè, Diego Thompson, David Charles Burr, Justin Halberda

**Affiliations:** 1 Department of Neuroscience, Psychology, Pharmacology, and Child Health, University of Florence, Florence, Italy; 2 Johns Hopkins University, Baltimore, MD USA; 3 Institute of Neuroscience, National Research Council, Pisa, Italy; 4 School of Psychology, University of Sydney, Sydney, NSW Australia

**Keywords:** Bayesian modelling, Object-based attention

## Abstract

Humans and non-humans can extract an estimate of the number of items in a collection very rapidly, raising the question of whether attention is necessary for this process. Visual attention operates in various modes, showing selectivity both to spatial location and to objects. Here, we tested whether each form of attention can enhance number estimation, by measuring whether presenting a visual cue to increase attentional engagement will lead to a more accurate and precise representation of number, both when attention is directed to location and when it is directed to objects. Results revealed that enumeration of a collection of dots in the location previously cued led to faster, more precise, and more accurate judgments than enumeration in un-cued locations, and a similar benefit was seen when the cue and collection appeared on the same object. This work shows that like many other perceptual tasks, numerical estimation may be enhanced by the spread of active attention inside a pre-cued object.

## Introduction

Perceiving the number of objects is a fundamental skill for many animal species (Agrillo & Bisazza, 2014; Dehaene, 2011), as well as for humans, including infants (Izard, Sann, Spelke, & Streri, 2009). What are the systems and faculties that support this ability? Intuitively, we cannot estimate the number of items in a visual collection unless we see the collection. But, will visual attention enhance or hinder estimation abilities? Perhaps neither, if focused attention is unnecessary for enumeration and number is extracted as an ensemble feature across the whole visual scene (Alvarez, 2011; Torralba, 2003; Torralba, Oliva, Castelhano, & Henderson, 2006). But when enumerating requires selecting a group of items from among the background, perhaps visual attention is necessary.

**Fig. 1:**
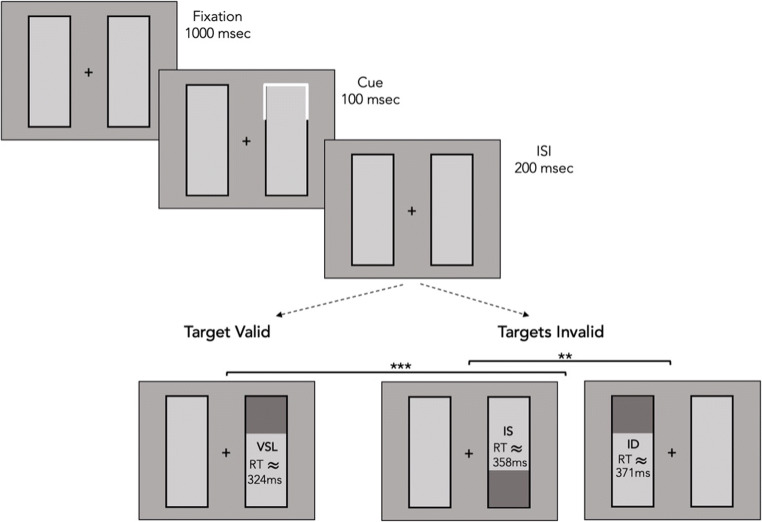
Schematic example of the typical sequence of Egly, Driver, and Rafal (1994). The target, illustrated in the bottom row of the figure, comprised filled squares: the valid target requires a shift in attention from the preceding cue; in contrast the invalid targets require a between- or a within-object shift from the cue. Shifting attention from a valid location to an invalid one results in a cost in terms of reaction time (RT). *ISI* interstimulus interval

Mechanisms of visual attention are employed to prioritize the processing of information in the environment at a particular moment. Past studies have shown that visual attention can be allocated either to a location in space or to an object, termed location-based attention or object-based attention respectively (Duncan, 1984; Egly, Driver, & Rafal, 1994; Posner, 1980). In a seminal study, Egly et al. (1994) used a double-rectangle display to demonstrate that attention can be delineated by the boundaries of objects, effectively spreading through attended objects. This has been shown by cueing one end of the two rectangles and then displaying a target either at the cued location (valid trials) or at one of three remaining locations (invalid trials). These locations include the other end of the attended rectangle (invalid same object, IS) as well as at the different ends of the unattended rectangle (invalid different object, ID). Faster responses to the target object at the uncued location of the same object compared to the uncued location on the different object has been interpreted as the result of spreading attention across the attended rectangle (Fig. 1). This explanation is based on the idea that all the two locations (i.e., same object different location, and different object) are equidistant from the cued location – thus responses on the attended object benefit from facilitated processing.

Despite the wealth of studies examining the role of location- and object- based attention on the detection or discrimination of visual stimuli (e.g., Brawn & Snowden, 2000; Shomstein & Behrmann, 2008), far less is known about the role of focusing attention on a specific location or object when extracting visual information about numerical quantity.

Recent evidence evaluating the connection between number, space, and attentional resources has demonstrated that depriving visual attentional resources in a concomitant visual or auditory dual task results in a higher cost in number representation in the small number range than in larger numerosities (Burr, Turi, & Anobile, 2010; Pomè, Anobile, Cicchini, Scabia, & Burr, 2019). Moreover, depriving attentional resources also distorts number mapping onto space. Usually, when asked to position numeric digits or clouds of dots appropriately on the “number line,” educated adults do so accurately (linearly). However, young children, children with dyscalculia, and unschooled adults with little math experience show strong non-linearities in number-line mapping, with the resulting number line taking on a logarithmic-like form (Ashkenazi & Henik, 2010; Booth & Siegler, 2006; Dehaene, Izard, Spelke, & Pica, 2008; Geary, Hoard, Nugent, & Byrd-Craven, 2008). If attentional resources are limited by a dual task, number-line mapping becomes logarithmic-like even in typical adults (Anobile, Cicchini, & Burr, 2012a), suggesting to some that depriving attention reveals the native “logarithmic” nature of number representation.

However, the fact that in these circumstances number representation follows a logarithmic form does not necessarily imply an intrinsic logarithmic representation of numerosity (Gallistel & Gelman, 1992; Karolis, Iuculano, & Butterworth, 2011). Several alternate explanations have been put forward, including the possibility that the nonlinearity results from a “central tendency of judgment” or “regression toward the mean” (Anobile, Turi, Cicchini, & Burr, 2012b), which applies to almost all properties – size, duration, speed, etc. (Hollingworth, 1910). This has recently been modelled within the Bayesian framework, where the mean across all experienced numbers is considered a *prior* (Anobile, Cicchini, & Burr, 2012a). As the effect of the prior depends on the relative reliability (reciprocal variance) of the sensory data, it will have more effect at high numerosities, as numerosity thresholds increase with numerosity. This will result in a compressive non-linearity, which can resemble a logarithmic transform (discussed in more detail later). Under conditions of uncertainty – such as under attentional load or with an unfamiliar numerical format – responses tend to be biased toward the mean of the stimulus distribution.

Further evidence that the non-linearity could reflect a dynamic process rather than a static non-linearity is that the response to the current trial correlates positively with the magnitude of the previous stimulus, suggesting that participants compute a weighted average of current and recent stimuli (Cicchini et al., 2014). This study reinforced the connection between the representation of number in space and the requirement of attentional resources, in showing that the dependency on previous stimuli was greater when attention had been deprived. However, only one study has so far investigated the role of increasing attentional engagement in a number task. The authors demonstrate that enhancement of attention through an alertness paradigm can improve the subitizing process (Gliksman, Weinbach, & Henik, 2016). To a lesser extent the improvement also occurred in the estimation range (from five to nine elements), particularly when elements were presented in a canonical arrangement.

The current study was designed to examine the role of attentional engagement in visual numerosity estimation, using a visual cuing paradigm. We had three specific aims: (1) to test the effects of enhancement of attention on numerosity perception, by directing attention to a previously cued object; (2) to study the effects of attentional engagement on the mapping of numbers onto space; and (3) to model the mapping effects within a Bayesian framework. Taking advantage of the contribution of both object- and location-based attention, we hypothesized that enumeration of quantity would benefit from focusing attention on a specific location, and that the advantages found there would spread to the whole object to which the cue had been presented. This should lead to faster, more accurate, and more precise estimates compared with when attention is directed to a different object. Moreover, we also explored the possibility that, especially in the condition of switching attention to a different object, the spatial representation of number would show a non-linear compression resulting from a central tendency like that described by previous studies for many sensory judgments, which we model within the Bayesian context.

## Methods

Fifteen subjects (mean age: 22.26 years; SD: 3.61) took part in the study. Informed consent forms were obtained from all the participants in accordance with the Declaration of Helsinki. Since 12 participants were recruited from Johns Hopkins University (Baltimore, MD, USA) and three from the University of Florence (Florence, Italy), the experimental protocol was approved by both the Institutional Review Board of Johns Hopkins University and the Italian regional ethics committee (*Comitato Etico Pediatrico Regionale—Azienda Ospedaliero-Universitaria Meyer*—Florence).

### Apparatus, stimuli, and procedure

Stimuli were generated using MATLAB software together with the Psychophysics Toolbox extensions (Kleiner, Brainard, & Pelli, 2007) and displayed on an LCD monitor driven by a Macintosh iMac computer (with a resolution of 1,920 x 1,080 pixels, refresh rate = 60 Hz). The subjects were seated approximately 50 cm from the screen and viewed the display binocularly. The displays comprised a pair of adjacent black rectangles oriented either vertically or horizontally with equal probability. Each rectangle (9.18° x 25.14°) was centered 6.65° from fixation. The fixation was a white cross (0.48° x 0.48°). The cue (three 9.18° x 0.28° white lines, overlapping one end of a rectangle) and the target, consisting of a collection of dots displayed in a circular region of 4°, were located at the end of the two rectangles. The background of all displays was gray. The default diameter of the dots was 0.25° and the maximum variability in size between dots was ± 31%.

In order to discourage reliance on a single continuous variable such as density or total surface area for numerical judgments (see, e.g., Feigenson, Libertus, & Halberda, 2013; Halberda & Feigenson, 2008; Libertus, Feigenson, & Halberda, 2011), we manipulated the non-numerical aspects of our stimuli so that on half of the trials the two arrays were equated for individual dot size (i.e., the average size of the dots in each collection was equal), and on the other half the cumulative surface area of dots was equated. The minimum distance between dots was 0.15°. Dot position was randomly determined with the constraint that dots never overlapped.

**Fig. 2:**
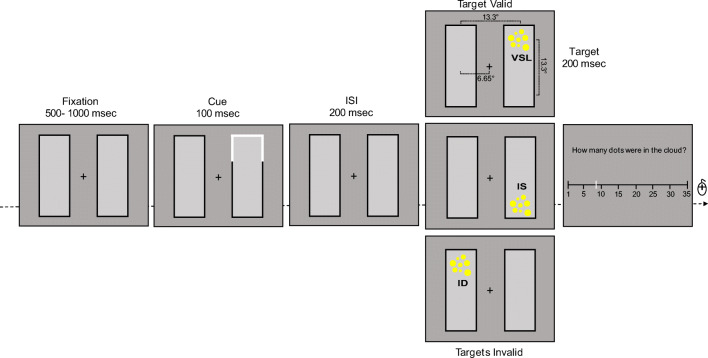
Example of a sequence of events within a trial (see also Fig. 1 for a schematic illustration of the original study by Egly et al., 1994). The target consisted of yellow dots presented either in the previously cued location (Target Valid, upper figure) or in an invalid location, which could be in the previously cued object (Target Invalid, middle figure) or in the other object (Target Invalid, lower figure). The collection of dots could never appear diagonal to the cue. After the presentation of the cloud of dots in one of the three possible locations, participants had to mouse click the corresponding perceived numerosity on a number line spanning from 1 to 35. *ISI* interstimulus interval

Each trial began with a fixation display comprising the central cross and two rectangles, which remained on for a random time from 500 to 1,000 ms, to avoid subjects predicting the onset of the cue. Following the fixation display, the cue was presented for 100 ms and then replaced by the fixation display for another 200 ms. The target comprised a cloud of yellow dots and was presented for 200 ms followed by a number line with extremes of 1 and 35 (with in-between number ticks spaced apart at equal distance), which extended for 42° (Fig. 2).

To initiate the session, participants pressed the space bar when ready. They were asked to fixate on the central cross throughout each trial, and to position and click a mouse pointer on the position of the number line corresponding to the estimated numerosity. Participants ran four sessions of 150 trials each, with numerosities ranging from 5 to 30, presented in random order.

Eye position was monitored visually by the experimenter during all sessions.

Each session comprised 75% valid trials, in which the cue and the target (cloud of dots) appeared in the same location (Valid Same Location, VSL); in the other 25% of trials the cue and the target did not appear in the same location: in 12.5% the target appeared in the cued object but at a different location (Invalid Same object, IS), and in the other 12.5% the target appeared in the un-cued object but equally distant from the cue (Invalid Different object, ID).

### Data analysis

The first 20 trials of each session were treated as training and excluded from the analyses. For each condition, responses that were more than two standard deviations above or below the mean were removed (less than 2% of the trials for each condition: 1.4%, 1.5%, and 1.8% of trials for VSL, IS, and ID, respectively).

All the analyses were conducted separately for the three different positions of cue and target: VSL, in which the target (cloud of dots) appeared at the same location of the cue; IS, in which the target appeared in the same cued object but at a different location; and ID, in which the target appeared in a different object than the cue.

We performed two types of analysis, a power fit and a Bayesian model of central tendency.

#### Power fit

In order to estimate the slope, intercept, and sigma of each subject's estimates we used a maximum-likelihood estimation approach, using an R-based package (PsiMLE; Odic, Im, Eisinger, Ly, & Halberda, 2016). Because of the unbalanced nature of our design (75% of the trials fell in the VSL condition and only 12.5% each in the IS and ID conditions), we took advantages of PsiMLE, which simultaneously estimates beta, intercept, and sigma, combining likelihood across different target values. PsiMLE allows for predicting responses to target values not presented or to restricted samples since it generates an entire probability distribution modeling participants’ internal representations (Odic et al., 2016)

This method maximizes the parameters that fit the normal distribution CDF (cumulative distribution function), with intercept (α), slope (*β*), and an extra parameter sigma (*σ*_*N*_) that describes the variability of the estimates of each dot quantity given the actual dot quantities (N) and participants' responses (R) with a likelihood function:

1 $$ \mathrm{L}\left(\upalpha, \upbeta, {\sigma}_N|\mathrm{N},\mathrm{R}\right)={\prod}_{\mathrm{k}=1}^{\mathrm{n}}\frac{1}{\sqrt{2\uppi {\left(\upalpha +\upbeta \ast \mathrm{N}\ast {\sigma}_N\right)}^2}}\exp \left(-\frac{{\left({\mathrm{R}}_{\mathrm{k}}-\upalpha +\upbeta \ast {\mathrm{N}}_{\mathrm{k}}\right)}^2}{2\ast {\left(\upalpha +\upbeta \ast {\mathrm{N}}_{\mathrm{k}}\ast {\sigma}_N\right)}^2}\right). $$

*N* corresponds to the number of dots presented (ranging from 5 to 30), α corresponds to the intercept of the linear regression, *β* corresponds to the slope, *σ*_*N*_ corresponds to the linearly increasing scalar variability (equivalent to coefficient of variation, CV), and *R* corresponds to participants’ number responses on the number line. PsiMLE simultaneously estimates all three parameters of interest. Using PsiMLE, we fit each participant’s estimates with the best fitting power function and obtained the corresponding three parameters of interest (exponent, scaling factor, and variability). The power function is given by:

2 $$ \mathrm{Y}=\upalpha {\mathrm{x}}^{\upbeta} $$

where α is the scaling factor, x is the actual numerosity tested, and β is the exponent. Our measure of estimation variability, *σ*_*N*_, correlated strongly with the more traditionally used CV measure (relation of *σ*_*N*_ to CV; *r* = 0.93, *p* < .001). Because the PsiMLE method yields better reliability in estimates (Odic et al., 2016), we used *σ*_*N*_ throughout our analyses. However, the pattern of results was very similar when analyzing CV with more standard techniques (standard deviation normalized by numerosity).

#### Bayesian model

To explore the possibility that the non-linear compression of the spatial representation of number could result from dynamic temporal context, producing regression to the mean, we modelled our number-line mapping with a Bayesian model similar to that used by Anobile, Cicchini, and Burr (2012a), which assumes that subjects base their performance on an estimate that combines both their sensory estimates and an *a priori* hypothesis about the stimulus.

Bayes’ rule states that:

3 $$ \mathrm{p}\left(\mathrm{R}|\mathrm{N}\right)\propto \mathrm{p}\left(\mathrm{R}\right)\mathrm{p}\left(\mathrm{N}|\mathrm{R}\right) $$

where *R* is the response and *N* is the numerosity of the stimulus. *p*(*N*|R) is termed the likelihood, *p*(R) the *prior*, and *p*(R|N) the *posterior*. We model likelihood with a Gaussian distribution centered on the stimulus, the width of which is estimated by the variability of the estimates (*σ*_*N*_), averaged over participants. The *prior* is also modelled as a Gaussian distribution centered on the mean of the stimulus range, with standard deviation free to vary to best fit the data.

**Fig. 3:**
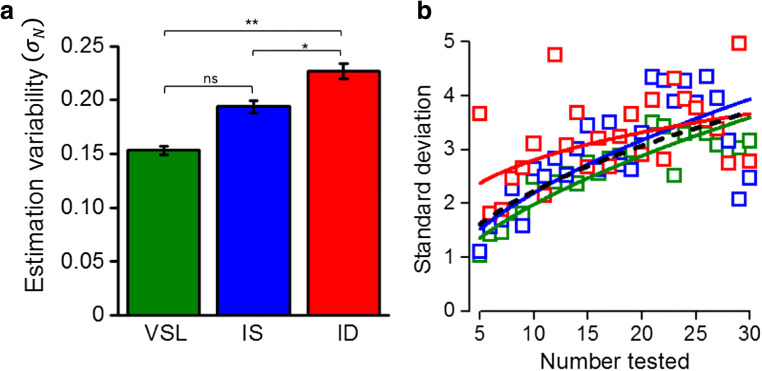
Precision analyses. (**A**) Estimation variability (*σ*_*N*_) obtained from the PsiMLE package for the three conditions: valid same location (VSL – green), invalid same object (IS – blue), and invalid different object (ID – red). Precision decreased as the attention was diverted from the cued location to the un-cued object, but there was no significant difference between the two valid cued conditions, same object or same location. Significance values refer to post hoc t-test comparisons (*p < 0.05, **p < 0.01, ns p > 0.05). (**B**) Standard deviations of responses as a function of physical number. The color-coded lines show the best-fitting power functions of the three conditions; the black dashed line is for the data pooled over conditions. The exponents for the four curves are: all data 0.48; VSL data in dark green 0.54; IS data in blue 0.53 and ID data in red are 0.24

Bayes’ Law states that the optimal combination of information is obtained by point-wise multiplication of the two Gaussian distributions:

4 $$ \varnothing \mathrm{p}\left(\mathrm{R}|\mathrm{N}\right)\propto \varphi \left(\mathrm{x}\right)\left(\upmu \mathrm{R},{\upsigma}_R\right)\varphi \left(\mathrm{x}\right)\left({\upmu}_{\mathrm{p}},{\upsigma}_p\right) $$

where *φ*(*x*) indicates the Gaussian function, the center of which is given by a weighted average of the centers of the likelihood and that of the prior. The resulting distribution is itself a Gaussian probability density function, the mean of which will be between the sensory estimate and the central prior:

5 $$ \hat{R}=\frac{\overline{P}{\sigma}_R^2+{N\sigma}_P^2}{\sigma_R^2+{\sigma}_P^2} $$

where $\hat{R}$ is the predicted response and $\overline{P}$ the mean of the prior (estimated to best fit the data).

6 $$ {\sigma}_R=k{N}^{\alpha } $$

where *k* is a constant and *α* the exponent, both estimated from the data. The constant *k* is given by the estimates of CV, separately for each condition, reported in Fig. 3. The extent to which the prior draws the results towards the mean depends on the relative widths of the prior and sensory likelihood functions. As the width of the sensory pdf increases with N, the effect will be stronger for large than for small numerosities, resulting in a compressive function (Fig. 5).

## Results

### Precision

We calculated the precision or variability (*σ*_*N*_) of the numerosity estimations for the three attentional conditions for each participant, using the PsiMLE package (Fig. 3A). Inspection of the figure revealed that precision decreased as attention was diverted from the cued to the un-cued object. In particular we found (post hoc comparisons) less precise estimation for invalid different object (ID) trials, compared with both the valid location trials (VSL: t = 3.65; p_tukey_ = 0.002) and the invalid same-object trials (IS: t = 2.44; p_tukey_ = 0.04). There was no significant difference between the two conditions within the same object (t = 1.20; p_tukey_ = 0.2), although cueing to the same position within the object (VSL) caused slightly (but non-significantly) lower thresholds than cuing different positions within the object (IS), 0.15 compared with 0.19.

We also examined how precision varied with numerosity (Fig. 3B). For this analysis, we did not use the PsiMLE package but calculated the SDs for the number line judgments. Because of the unbalanced design of the experiment (only a few trials in the invalid conditions), we collapsed data over participants, and performed a two-way ANOVA, with condition and numerosity as factors. Both main factors were significant (F(1,26) = 6.74, p = 0.01; F(25,26) = 2.02, p = 0.04 for condition and numerosity, respectively), but the interaction was not (F(25,26) = 0.27, p = 0.99). This separate analysis as a function of numerosity confirms the PsiMLE analysis, showing a difference between the conditions. From inspection, the unattended condition clearly has the higher thresholds.

The color-coded lines through the data are best-fitting power functions, following Eq. 2. All three conditions follow a compressive non-linearity, with exponents of 0.54, 0.53, and 0.24 for conditions VSL, IS, and ID, respectively. However, as there was no significant interaction in the ANOVA, and the number of trials is very small in the unattended condition (12.5% total), there is no evidence that the dependency on numerosity is actually different. We therefore fit all the data together, collapsing over numerosity to get more robust estimates of standard deviation, and fit those data with a power function. This fit is shown by the black curve, and has an exponent of 0.48, similar to the two attention conditions. This implies that precision thresholds do not actually scale with numerosity, as Weber’s Law would imply, but scale with the square-root (exponent 0.5) of numerosity, as has been found in the past with number-line tasks (Cicchini et al., 2014). We will use this relationship with numerosity to model the Bayesian predictions in Fig. 6.

### Accuracy of number line

**Fig. 4:**
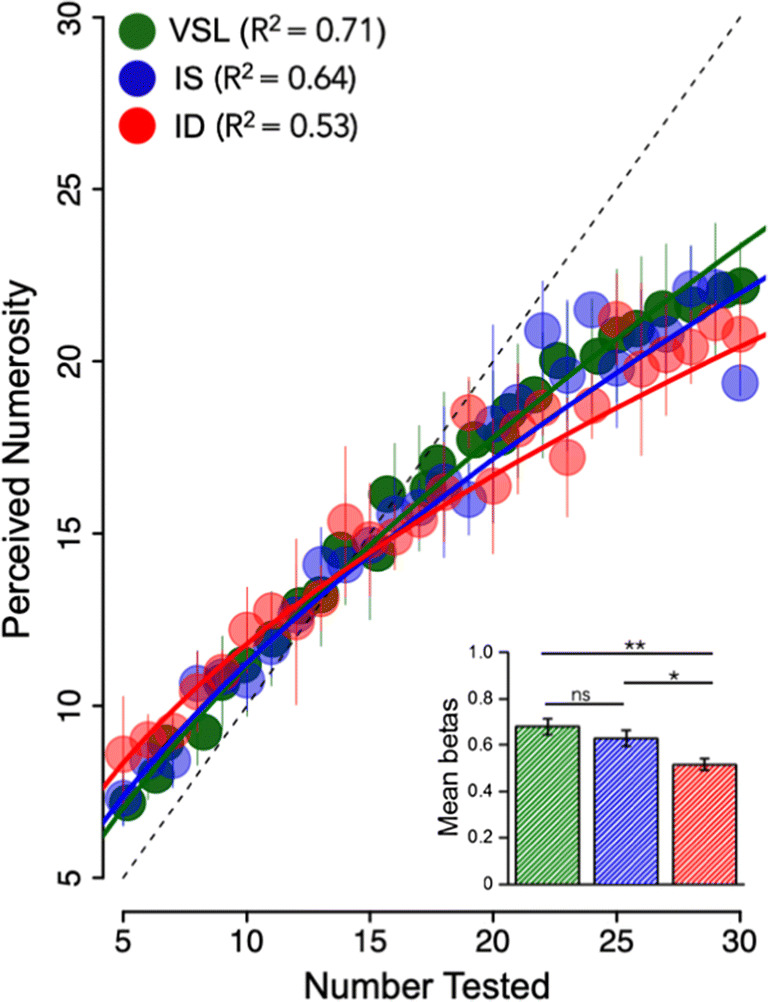
Relationship between the presented numerosity and average estimates, estimated separately for the three conditions (valid same location in green, invalid same object in blue, and invalid different object in red). Continuous lines represent the power-fitting function (*β*). Thin color-coded vertical lines represent the standard deviations. The small inset on the bottom right represents the mean betas for the three conditions, respectively. Significance values refer to post hoc comparisons (*p < 0.05, **p < 0.01, ns p > 0.05)

We next examined the average accuracy of number-line mapping for the three different conditions. Figure 4 shows the results, plotting average numerosity estimates against actual numerosity, averaged over all subjects. Data from each condition are fit with a power function (Eq. 2), pooling together all participants. The mean exponents of the power function (*β*) of Eq. 2 (obtained from fitting each participant’s estimates with the best fitting power function using PsiMLE) were, respectively, 0.68 (±0.03) for the VSL condition in green; 0.63 (±0.03) for the IS condition in blue; and 0.51(±0.02) for the ID condition in red (see inset bar graph). The exponents reveal the non-linearity of the relationship, with the VSL condition being the more linear while the other two conditions demonstrate progressively increasing compressive non-linearities.

For every subject, the non-linear component was far larger in the ID condition (different object) than in the IS condition (same object). Post hoc comparisons showed a significant difference in non-linearity between the condition in which the target appeared in the cued object but in a different location compared to the condition in which the target appeared in a different object (t = 2.57; p_tukey_ = 0.03), and a further significant spatial cuing effect with VSL trials being more linear than ID (t = 3.7; p_tukey_ = 0.002), but not significantly more linear than IS trials (t = 1.13; p_tukey_ = 0.49).

Overall, the measurements of precision and accuracy confirm the hypotheses that estimation of numerosity is improved by the spread of attention within the cued object, at the expense of the object outside the focus of attention.

### Regression to the mean

**Fig. 5:**
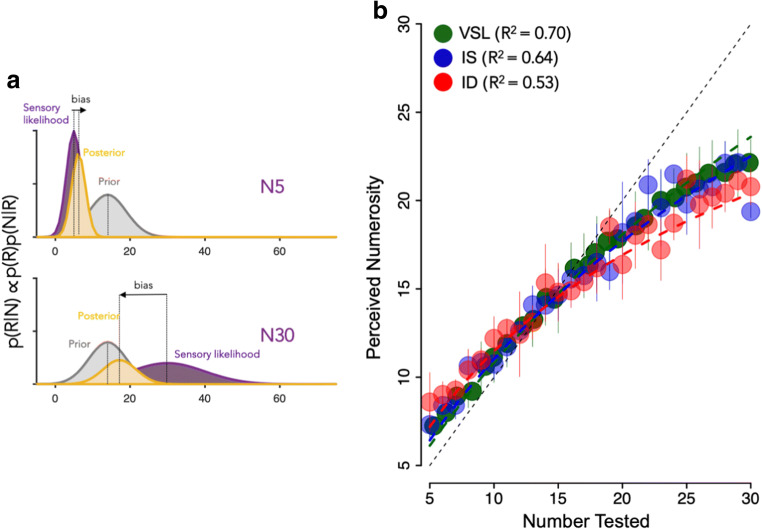
Illustration of the central-tendency model of non-linear mapping. (**A**) Probability density functions for *likelihood*, *prior*, and *posterior* (Eq. 3), for two physical displays of 5 or 30 dots to be mapped onto a 1–75 number line. For all three number lines, the prior is a Gaussian probability density function centered at 15 dots on the number line with a standard deviation of 5 (determined by best fit to data). The likelihood was also Gaussian, centered at the physical number *L*, with a standard deviation of 3 (determined by best fit to data). The posterior is the product of the sensory likelihood and the prior. If the prior is closer to the center of the test range, the posterior will be biased towards the center of the distribution. The strength of the bias depends on the relative uncertainty of likelihood and prior. As the standard deviation of the likelihood for larger magnitudes increases, the bias towards the prior also increases. (**B**) Data from Fig. 4, with the simulations shown by dashed curves. Thin color-coded vertical lines represent the standard deviations

Following the procedure of Anobile et al. (2012), we fit our data with the Bayesian model of central tendency (Eq. 3), where the *posterior* probability of a particular response is given by the normalized product of the sensory data (the likelihood distribution resulting from a given number of dots, estimated from the data) and the “central tendency” *prior*, which draws the sensory estimates towards the center. Curves in Fig. 5B are the best fits assessed by minimizing *R*^*2*^, the ratio between the Explained Sum of Squares and the Residual Sum of Squares. Best fits of the data were obtained with prior centered at 15, near the mid-point of the stimulus range (5–30). Overall the curves show that the Bayesian model for the three number lines clearly captured the pattern of results (0.53 < R^2^ < 0.70). For the conditions of VSL and IS, the number line is quite linear (dark green and blue dashed lines), while for ID trials (red line) the mapping shows a clear compressive non-linearity, as previously observed (Anobile et al., 2012a).

**Fig. 6:**
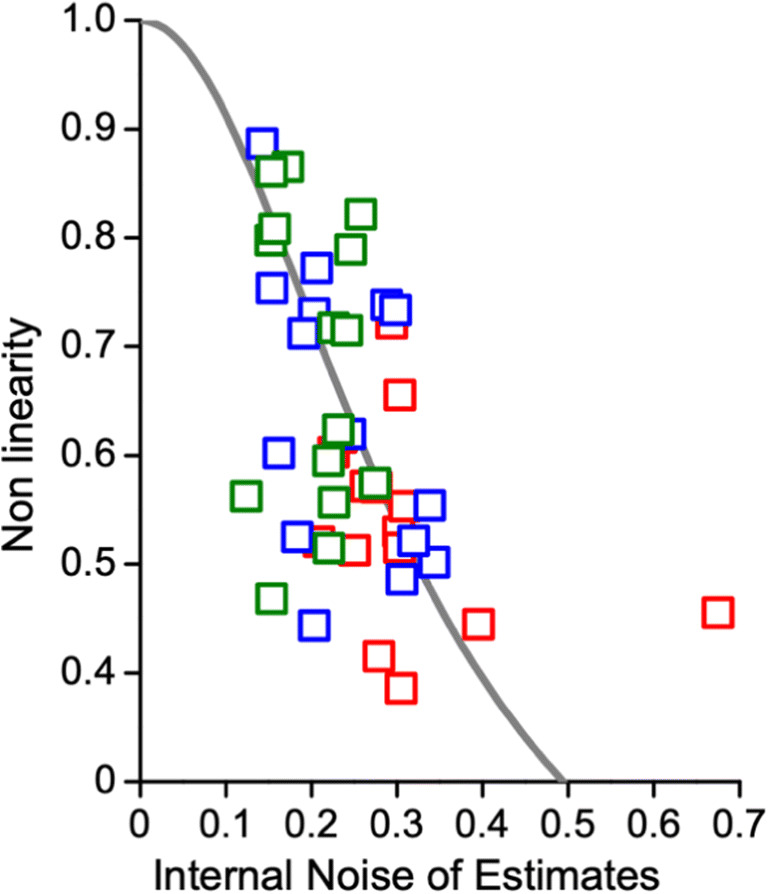
Non-linearity index plotted against a measure of internal noise of estimation. The gray curve shows Bayesian model predictions (see Eq. 7); color-coded squares refer to the different condition tested (valid same location green; invalid same object blue, and invalid different object red)

As the Bayesian model of central tendency predicts that the magnitude of mapping distortion should depend on the level of sensory noise, we measured the correlation between the non-linearities assessed by the fit to the power function (*β* of Eq. 2) and individual internal noise of estimates. This measure was obtained by dividing the responses by the actual number tested and then computing the standard deviation (SD) for the three conditions separately. Figure 6 shows the relationship between non-linearity and discrimination thresholds. As predicted, participants with higher discrimination thresholds also had higher non-linearities (r = 0.45, *p* = 0.002).

We also show the prediction of an ideal observer model that uses a prior standard deviation of 17 dots, derived from the averaged stimuli intensity. The Bayesian model fit, with one degree of freedom (width of the prior), showed a R^2^ of 0.15. We also performed the same analysis on our measure of estimation variability (*σ*_*N*_), and the pattern of results looked the same. To compute the fit, we used the model of Cicchini, Anobile, and Burr (2018), of an ideal observer, which blends current noisy sensory information with a central prior.

7 $$ {W}_L=\frac{\sigma_P^2}{x^2\ast {WF}^2+{\sigma}_P^2} $$

### Reaction times

Finally, since the previous literature on location- and object-based attention has focused on the differences in reaction times (RTs) between the condition tested (suggesting a RT advantage for target presented in the same previously cued location or the same previously cued object compared to the uncued object), we also analyzed RTs to respond on the number line. RTs of less than 150 ms were not included in the analysis (less than 2% of the trials).

**Fig. 7:**
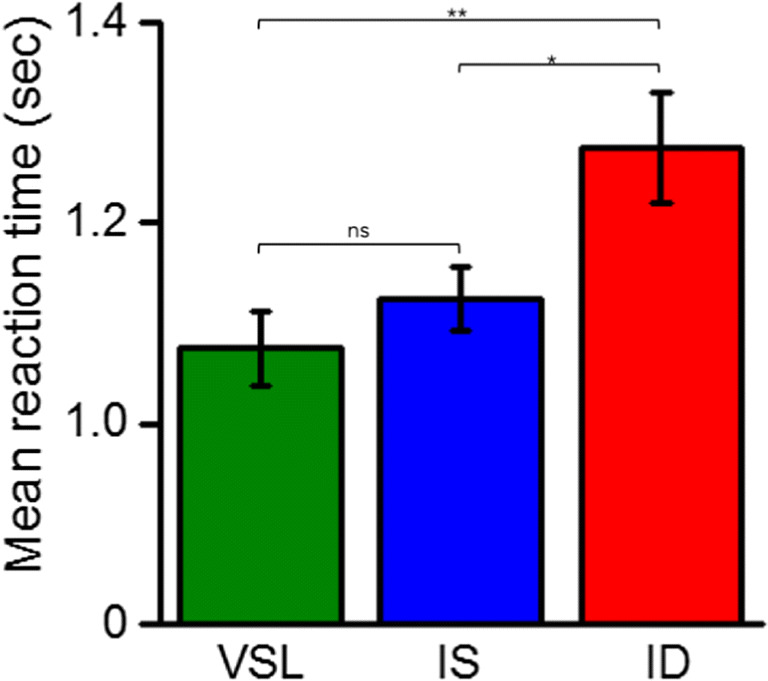
Mean reaction times. Averaged reaction times calculated for each participant and each condition (Valid same location in green; invalid same object in blue; invalid different object in red). Color-coded dots represent the mean reaction times each subject each condition. Significance values refer to post hoc comparisons (*p < 0.05, **p < 0.01, ns p > 0.05)

Data in Fig. 7 show averaged RTs calculated for each participant and each condition. Post hoc comparisons showed a significant 200-ms cueing effect (t = 3.44; p_tukey_ = 0.004), with valid same-location RTs (1.07; 0.14) being faster than invalid different object (1.27; 0.20). The cue also led to faster responses when the target appeared at the cued object (IS) than at the un-cued object (ID) (t = 2.58; p_tukey_ = 0.03), with a 140-ms advantage. These results suggest that estimation of numerosities suffers from the shift of attention from one cued object to another un-cued object, leading to slower RTs in computing the estimation (same-object advantage). Moreover, a comparison between the VSL and IS revealed that participant RTs were not significantly faster when the target appeared at the cued location compared to the cued object (t = -0.85; p_tukey_ = 0.67). These results reinforce the precision and accuracy results in suggesting that both location-based and object-based cues are informative, and that attention spread from the cued location to the whole object.

## Discussion

The present study examined the role of attention in the enumeration process. Previous studies have shown that when attention is disengaged from a numerosity task, the subitizing process is impaired. Much less impairment was found in the estimation process. Here, we examined whether presentation of a visual cue that increases attentional engagement in a given task can facilitate the estimation process, leading to a less compressive representation of number in space compared to when attention is diverted elsewhere. The results revealed that enumeration of a collection of dots in the location previously cued led to more precise and more accurate judgments than enumeration in uncued locations. In particular, the visual cue facilitated the estimation process when both the cue and the target were presented in the same previously cued object.

Since much literature on object- and location-based attention has examined the effects of attention by measuring RTs, we also calculated the time participants took to report their responses on the number line. Consistent with previous findings, RTs were faster when the target appeared in the previously cued location compared with uncued locations, with an advantage also when the target was presented in the cued object compared with the uncued object.

Findings of previous studies on the contribution of object- and location- based attention pointed out two main effects: a spatial cueing effect and a same-object advantage (the first referring to advantages in detecting the target when it appears in the cued location; the latter referring to advantages in detecting the target when it appears in the cued object rather than the uncued object). Here we first demonstrated that these results generalized to the extraction of numerosity. We also demonstrated that numerosity benefits from object-based attentional resources. In particular, the same attentional advantage found here for cuing within the object as cuing the precise location suggests that attention to number spreads from the cued location to the whole cued object. As previously mentioned, studies on the contribution of attention to numerosity perception have mainly focused on the effect of depriving attentional resources in a number task. Enhancing rather than depriving attention can affect enumeration within the subitizing range. Gliksman et al. (2016) found that cued arrays within the subitizing range were enumerated faster than uncued arrays, indicating that subitizing is an attention-dependent process and can be manipulated through enhanced alertness. This alerting effect, when enumerating arrays within the subitizing range, has also been found in individuals diagnosed with developmental dyscalculia, although such cuing was unable to expand their smaller-than-normal subitizing range (Gliksman & Henik, 2018). Here we expand these results by finding that, also within the estimation range, previously cued arrays are enumerated faster, more precisely, and more accurately than uncued arrays, indicating that estimation processes are facilitated by inducing a manipulation that increases attentional engagement during an enumeration task.

When asked to position the perceived numerosity of clouds of dots on a number line, humans normally do so accurately, that is, linearly. However, when attentional resources are diverted by a concurrent demanding conjunction task, the judgments become distinctly non-linear, which is well described by a logarithmic relationship (Anobile, Cicchini, & Burr, 2012a). This suggests that both linear and compressed maps can coexist, and the use of one or the other may be due to a variety of task-driven strategic factors. It has been suggested that the neural substrate underlying the logarithmic mapping of number may reflect the bandwidth of neurons selective to number, as in both non-human and human primates neural responses in the intraparietal sulcus show a logarithmic-like tuning, with bandwidth proportional to preferred number (Nieder, 2005; Nieder & Merten, 2007; Piazza, Izard, Pinel, Le Bihan, & Dehaene, 2004), consistent with a pre-attentive logarithmic mapping onto the number line.

However, another realistic possibility is that the compression may reflect a “central tendency of judgments,” a long-standing notion (Hollingworth, 1910). That numerosity may be subject to the central tendency is further support for the notion of number being a primary visual attribute (Burr & Ross, 2008). Jazayeri and Shadlen (2010) have recently revived the concept of central tendency in Bayesian terms, where the mean becomes the *prior*, which combines with the sensory likelihood to produce the *posterior,* which will be biased towards the mean. Given that the likelihood is essentially the product of the Weber fraction and dot number, and Weber fraction is fairly constant, the likelihood is much broader at the higher than lower number range. Thus, the higher range will be more drawn towards the mean than the lower range, and this will result in the compressive non-linearity that is observed in Fig. 5, and well fitted by a power function with exponent less than one. It is also well approximated by a logarithmic function (Siegler & Opfer, 2003; Stanislas Dehaene et al., 2008). Here we modelled our number-line data with a simple Bayesian model that predicted the compressive shape, and fitted the data well, accounting for about 60% of the variance.

What purpose does the prior – and central tendency in general – serve? As others have argued, a prior based on the statistics of the sensory events can improve performance – measured as the sum of total error – at the expense of reducing veridicality (see Jazayeri & Shadlen, 2010). Effectively, under conditions of uncertainty, performance can be improved by considering the past history of events. This could explain why under the condition of the invalid different object, given that the precision is lower, the prior becomes more effective (as it is the relative widths of prior and sensory likelihood that determine the extent of central tendency).

Each of the aspects we have noted (e.g., the benefits of attention, both at a location and within an object; the pull of a central tendency on number responses) might function either at the level of number perception (e.g., the extraction algorithms that take visual evidence as input) or number representation (e.g., the resulting Gaussian curves that represent number along a mental number line). Indeed, there are likely to be effects at each of these levels of processing (e.g., perhaps the effects of attention are primarily in number perception (i.e., extraction), while the effects of a central tendency are at the level of number representations). This will be an interesting area for future research.

It is clear that number, space, and attention are interconnected. A growing body of evidence links the ontogenetically inherited nonverbal system with the culturally invented and linguistically mediated number code (Feigenson et al., 2013). Number acuity, which improves during development (Halberda & Feigenson, 2008), correlates with formal mathematics achievement (Anobile, Stievano, & Burr, 2013; Mazzocco, Feigenson, & Halberda, 2011) and predicts math skills years later (Halberda, Mazzocco, & Feigenson, 2008). Conceptions of how numbers map onto space develop during school years (Booth & Siegler, 2006; Siegler & Booth, 2004; Siegler & Opfer, 2003); kindergarten children represent numbers in space in a compressed, seemingly logarithmic scale (e.g., placing the number 10 near the midpoint of a 1–100 scale). The scale becomes progressively more linear over the first 3 or 4 years of schooling. Interestingly, dyscalculic children (those who suffer from a specific mathematical learning disability) show poor number acuity (Halberda et al., 2008; Piazza et al., 2010) and a more logarithmic representation of the number line than controls (Ashkenazi & Henik, 2010; Geary, Hoard, Byrd-Craven, Nugent, & Numtee, 2007; Geary et al., 2008). Other studies have shown that, along with deficits in numerical processing, dyscalculics also show deficits in attention (Ashkenazi & Henik, 2010), and there is a clear interplay between attention and math skills (Anobile et al., 2013). It would be interesting to study the relationship between attention enhancement and math abilities.

To conclude, the present study revealed that the estimation process could be facilitated by inducing a manipulation that increases attentional engagement during an enumeration task. Research focusing on a mechanism that can enhance rather than deprive attentional resources could prove helpful when considering rehabilitation in conditions such as dyscalculia. In particular, future research can examine whether procedures that act to increase attention can also improve the ability to map number into space in dyscalculic populations.
